# Editorial

**DOI:** 10.1007/s11618-023-01152-0

**Published:** 2023-04-21

**Authors:** Marcus Hasselhorn

**Affiliations:** DIPF | Leibniz-Institut für Bildungsforschung und Bildungsinformation, Rostocker Str. 6, 60323 Frankfurt am Main, Deutschland

Seit wenigen Jahren küren die Herausgeberinnen und Herausgeber der Zeitschrift für Erziehungswissenschaft in jedem Jahr einen Editors’ Choice Beitrag, bei dem die Erstautorin bzw. der Erstautor bei Annahme des Beitrags noch nicht das 40. Lebensjahr vollendet hat und sich auch noch nicht auf einer unbefristeten Professur befindet. Editors’ Choice meint dabei, dass die ZfE-Verantwortlichen diesen Beitrag als den besten des Jahres unter der federführenden Verantwortung einer Person aus der noch jungen Generation von Forschenden halten. Im Sommer letzten Jahres haben wir bei der ZfE alle für diese Kategorie formal in Frage kommenden Beiträge aus dem Jahr 2021 zusammengestellt und eine elektronisch geheime Abstimmung in unserem Herausgeber-Team vorgenommen. Unverhoffte technische Probleme machten es erforderlich, dass wir im Dezember die Wahl wiederholen mussten. Dabei ergab sich ein einstimmiges Votum: Der Beitrag „*Learning during COVID-19: The role of self-regulated learning, motivation, and procrastination for perceived competence*“ von Elisabeth R. Pelikan et al. wurde ausgewählt. Bereits innerhalb der ersten ca. eineinhalb Jahre nach Veröffentlichung des Beitrages wurde er in der einschlägigen Literatur mehr als 120 Mal zitiert, was zeigt, dass er nicht nur uns als ZfE-Verantwortliche überzeugt. Unser herzlichster Glückwunsch an Frau Pelikan und das Team der weiteren Autorinnen und Autoren aus Wien.

Eine Neuigkeit haben wir auch in eigener Sache zu vermelden. Seit Jahresbeginn wird unser Team der ZfE-Herausgebenden durch Prof. Dr. Steffen Hillmert von der Universität Tübingen bereichert. Herzlich willkommen!

Auch freuen wir uns, dass wir mit dem Ihnen vorliegenden ersten Heft 2023 bzw. des 26. Jahrgangs der Zeitschrift für Erziehungswissenschaft unser 25jähriges Jubiläum verkünden dürfen. Bereits in unserem ersten Erscheinungsjahr, 1998, haben wir 4 Hefte mit Themenschwerpunkten, wie „Medien“, „Allgemeine Erziehungswissenschaft und andere Teildisziplinen“, „Gerechtigkeit“, „Arbeitsgesellschaft und Bildung im Wandel“, sowie weiteren Beiträgen zu den Themen „Jugend“, „Historische Anthropologie“, „Lernen“, „Klaus Mollenhauer zum Gedenken“ und „Schulforschung“ publizieren können. Schon in der damaligen Themenauswahl zeigte sich das Selbstverständnis der ZfE, repräsentatives wissenschaftliches Fachorgan für die gesamte Breite erziehungs- und bildungswissenschaftlicher Forschung zu sein.

Das vorliegende Heft ist eines ohne Schwerpunktthema. Die zehn Beiträge decken ein breites Spektrum erziehungswissenschaftlicher Arbeitsweisen, Ansätze und Themen ab. Der erste Beitrag von Madeleine Müller und Bernadette Gold beschäftigt sich mit dem professionellen Erkennen und Analysieren relevanter Situationen als eine grundlegende Voraussetzung für das professionelle Handeln einer Lehrkraft. Deren Erfassung erfolgt oft mit offenen oder geschlossenen videobasierten Verfahren. An einer Stichprobe von mehr als 300 Lehramtsstudierenden wurden bei Verwendung eines identischen Videostimulus statistische Zusammenhänge der beiden Erfassungsformate geprüft. Die Ergebnisse weisen darauf hin, dass die beiden Erfassungsformate unterschiedliche Konstrukte messen, die allerdings in beiden Fällen kein eindeutiges Zusammenhangsmuster mit dem professionellen Wissen über Klassenführung aufwies.

Mit der Frage der inklusionsbezogenen Fortbildungsmotivation von Lehrkräften setzt sich der zweite Beitrag von Tobias Wächter und Julia Gorges auseinander. Geprüft wird die Annahme, dass eine positive Einstellung zu schulischer Inklusion eine zentrale Rolle für die engagierte Teilnahme an entsprechenden Fortbildungen spielt. Die Einstellung zu Inklusion von 166 Lehrkräften wurde im Zusammenhang mit einer bevorstehenden Fortbildung zur Kooperation an inklusiven Sekundar- und Gesamtschulen erfasst. Unter Nutzung von Mehrebenen-Analysen kann gezeigt werden, dass Einstellung zu Inklusion die fortbildungsbezogene Erfolgserwartung sowie die Teilnahme vorhersagt. Außerdem scheint eine stark ausgeprägte persönliche Selbstwirksamkeitserwartung teilnahmehinderliche Wertüberzeugungen reduzieren zu können.

Im dritten Beitrag von Andrea Burda-Zoyke, Robert Jahn, Thomas Driebe und Mathias Götzl geht es auch um inklusionsbezogene Einstellungen. Thematisiert werden Einflussfaktoren auf die entsprechenden Einstellungen bei Lehrkräften an berufsbildenden Schulen. 662 Lehrkräfte an beruflichen Schulen aus vier Bundesländern wurden zu ihren Einstellungen zur Inklusion befragt. Berufliche und private Erfahrungen mit Menschen mit besonderen Bedürfnissen, Erfahrungen in inklusiven Klassen und die inklusionsbezogene Qualifizierung erwiesen sich als Prädiktoren der persönlichen Bereitschaft als eine Dimension der Einstellungen. Zudem zeigt die inklusionsbezogene Selbstwirksamkeitserwartung einen positiven Effekt auf die Einstellungen.

Mit Schülerinnen und Schülern teilqualifizierender Berufsfachschulen beschäftigt sich der vierte Beitrag von Sylvia Rahn und Christoph Fuhrmann. Diese gehen mit der Erwartung in die beruflich teilqualifizierenden höheren Berufsfachschulen, ihr Schulabschlussniveau und ihre Übergangschancen in eine duale Berufsausbildung verbessern zu können. Allerdings ist die Effektivität der zweijährigen beruflich teilqualifizierenden höheren Berufsfachschulen umstritten. Auf der Basis einer regionalen Panelstudie wurde deshalb untersucht, welche Ausbildungschancen die beruflich teilqualifizierenden höheren Berufsfachschulen eröffnen und ob die Bildungsgänge im Berufsfeld Wirtschaft und Verwaltung tatsächlich den Weg in die Ausbildung zu kaufmännischen Fachangestelltentätigkeiten ebnen. Im Beitrag kann belegt werden, dass die Absolvent*innen der beruflich teilqualifizierenden höheren Berufsfachschulen im Vergleich mit den Direktabgänger*innen der Sekundarstufe I mit mittlerem Abschluss ihre Chancen auf dem Ausbildungsmarkt tatsächlich verbessern können. Zugleich verlassen jedoch etwa 20 % der Schülerinnen und Schüler den Bildungsgang vorzeitig.

Doris Holzberger, Janina Täschner und Delia Hillmayr geben im fünften Beitrag dieses Heftes einen systematischen Überblick über vorliegende Metaanalysen zum Zusammenhang zwischen Elternbeteiligung und Schulerfolg. Anhand der Methode des Second-Order Reviews wurden 21 Metaanalysen mit insgesamt 1268 zugrundeliegenden Primärstudien ausgewertet. Es zeigten sich größere Zusammenhänge zwischen Elternbeteiligung und Schulerfolg für Familien mit einem höheren sozioökonomischen Status. Moderatoreffekte für den Migrationshintergrund oder die ethnische Herkunft blieben aus. Uneinheitlich waren die Befunde mit Blick auf die Altersgruppen.

Im sechsten Beitrag von Markus Spilles, Christian Huber, Philipp Nicolay, Johannes König und Thomas Hennemann werden Schulkind-Dyaden in der Grundschule analysiert, um den Zusammenhang zwischen Regeleinhaltung und Lehrerfeedback und der sozialen Akzeptanz von Grundschulkindern zu klären. Vier Forschungsfragen werden dabei betrachtet: (1) Gibt es einen Zusammenhang zwischen Regeleinhaltung und sozialer Akzeptanz? (2) Wird der Zusammenhang von Regeleinhaltung und sozialer Akzeptanz durch die Regeleinhaltung des urteilenden Kindes moderiert? (3) Gibt es einen Zusammenhang zwischen Lehrkraftfeedback und sozialer Akzeptanz? Und (4) Wird der Zusammenhang von Regeleinhaltung bzw. Lehrkraftfeedback und sozialer Akzeptanz durch die Klassenstufenzugehörigkeit moderiert? Querschnittsdaten von fast 1000 Kindern aus 50 zweiten, dritten und vierten Klassen wurden einer logistischen Mehrebenen-Regressionsanalyse mit Random-Intercept-Modellen mit Berücksichtigung der kreuzweisen Nestung von insgesamt 17.409 Schulkind-Dyaden unterzogen. Die Ergebnisse bestätigen, dass die soziale Akzeptanz eines Kindes sowohl mit der Regeleinhaltung (Frage 1) als auch mit dem Lehrkraftfeedback (Frage 3) zusammenhängt. Schulkinder, die sich selbst gut an die Klassenregeln halten, neigen dazu, auch solche Mitschülerinnen und Mitschüler zu wählen, bei denen sie ebenfalls eine hohe Regelkonformität einschätzen (Frage 2). Der Zusammenhang zwischen sozialer Akzeptanz und Regeleinhaltung bzw. Lehrkraftfeedback scheint sich mit zunehmender Jahrgangsstufe zu steigern (Frage 4).

Der siebte Beitrag von Sarah-Lisa Hecker, Stephanie Falkenstern, Svenja Lemmrich, Timo Ehmke, Barbara Koch-Priewe, Anne Köker und Udo Ohm befasst sich mit Lehrkräfte-Expertise im Bereich Deutsch als Zweitsprache (DaZ). Ausgehend von Annahmen aus der Expertiseforschung werden die Testergebnisse von knapp 300 angehenden und praktizierenden Lehrkräften genutzt, um unter aktiver Beteiligung von einschlägigen Fachexpertinnen und Fachexperten mithilfe der Bookmark-Methode theorie- und datenbasiert Schwellen von Kompetenz zu ermitteln. Die hergeleiteten Kompetenzniveaus werden anhand eines Beispiel-Testitems inhaltlich beschrieben.

Die Frage, was Bildungswissenschaften sind, wirft der achte Beitrag von Felix Schreiber und Colin Cramer auf. Anhand eines inhaltsanalytischen Vorgehens (Conceptual Systematic Review) werden die in der einschlägigen Literatur vorfindbaren Auffassungen von Bildungswissenschaft(en) hinsichtlich Häufigkeit ihres Auftretens und ihrer Zusammenhänge analysiert. Identifiziert werden 17 unterschiedliche Auffassungen von Bildungswissenschaft(en). Dominierend sind dabei Auffassungen, diese als ‚Komponente der Lehrer:innenbildung‘ oder als ‚(Neu‑)Bezeichnung für Pädagogik und Erziehungswissenschaft‘ zu definieren. Diskursiv sind auch Auffassungen von Bildungswissenschaft(en) im Sinne einer ‚kompetenzorientierten Neuordnung‘ und einer ‚reflexiven Ausweitung‘ prominent. Herausgearbeitet werden drei Zusammenhangs-Cluster, die inhaltlich entweder auf die disziplinäre Struktur der Erziehungswissenschaft, die institutionellen Aufgaben der Lehrerbildung oder ein methodisch engeres Forschungsprogramm verweisen.

Mit der Rolle migrationsbezogener Überzeugungen pädagogischer Fachkräfte für die Qualität frühpädagogischer Praxis beschäftigt sich der neunte Beitrag dieses Heftes von Csaba Kurucz, Axinja Hachfeld, Simone Lehrl und Yvonne Anders. Die Autor*innen untersuchen, ob die migrationsbezogenen Überzeugungen im Zusammenhang mit der in ihren Kindertageseinrichtungen beobachteten sprachspezifischen Prozess- und Strukturqualität stehen. Als migrationsbezogene Überzeugungsfacetten wurden die multikulturellen und assimilativen Überzeugungen berücksichtigt. Die sprachspezifische Prozess- und Strukturqualität wurde mithilfe einschlägiger Subskalen eingeschätzt. Die multikulturellen und assimilativen Überzeugungen der frühpädagogischen Fachkräfte gingen neben ausgewählten Struktur- und Fachkraftmerkmalen getrennt in Strukturgleichungsmodelle ein. Im Gegensatz zu den assimilativen Überzeugungen erwiesen sich die multikulturellen Überzeugungen der frühpädagogischen Fachkräfte als bedeutsam für einige der Qualitätsmaße.

Der abschließende zehnte Beitrag thematisiert die Auswirkungen der Covid-19-Pandemie auf die Fallarbeit der Jugendämter. Pascal Bastian, Megan Benoit, Katharina Freres und Jana Posmek führten dazu Telefoninterviews mit den Fachkräften zweier Jugendämter durch. Aus einer relationalen Perspektive im Sinne Bruno Latours wurden die Interviews kontrastiv ausgewertet. Dabei wurden zunächst Verschiebungen des Netzwerkes sichtbar, in dem die Fälle üblicherweise bearbeitet werden. Diese Transformationen wurden auf der Grundlage der Interviews als krisenhaft erlebte Einschränkungen der üblichen Handlungspraxis gedeutet. In den analysierten Daten lassen sich Handlungsweisen rekonstruieren, die sich als Umgang mit dieser Krise fassen lassen. Die eigentliche Krise scheint nicht die abstrakte Vorstellung einer Viruspandemie darzustellen, sondern vor allem das Wegbrechen von hergebrachten Fallarbeitsgewohnheiten.

